# α-Ketoglutarate inhibits autophagy

**DOI:** 10.18632/aging.102001

**Published:** 2019-06-07

**Authors:** Elisa Elena Baracco, Francesca Castoldi, Sylvère Durand, David P. Enot, Jelena Tadic, Katharina Kainz, Frank Madeo, Alexis Chery, Valentina Izzo, Maria Chiara Maiuri, Federico Pietrocola, Guido Kroemer

**Affiliations:** 1 Centre de Recherche des Cordeliers, INSERM, Sorbonne Université, Université Paris Descartes, Université Paris Diderot, "Metabolism, Cancer and Immunity", Paris 75006, France; 2 Metabolomics and Cell Biology Platforms, Gustave Roussy Cancer Campus, Villejuif, France; 3 Institute of Molecular Biosciences, University of Graz, NAWI Graz, Graz, Austria; 4 Division of Endocrinology and Diabetology, Department of Internal Medicine, Medical University of Graz, Graz, Austria; 5 BioTechMed Graz, Graz, Austria; 6 Institute for Research in Biomedicine, Barcelona, Spain; 7 Pôle de Biologie, Hôpital Européen Georges Pompidou, AP-HP, Paris, France; 8 Karolinska Institute, Department of Women’s and Children’s Health, Karolinska University Hospital, Stockholm, Sweden

**Keywords:** acetyl CoA, aging, cell death, Krebs cycle, metabolomics, mitochondria

## Abstract

The metabolite α-ketoglutarate is membrane-impermeable, meaning that it is usually added to cells in the form of esters such as dimethyl −ketoglutarate (DMKG), trifluoromethylbenzyl α-ketoglutarate (TFMKG) and octyl α-ketoglutarate (O-KG). Once these compounds cross the plasma membrane, they are hydrolyzed by esterases to generate α-ketoglutarate, which remains trapped within cells. Here, we systematically compared DMKG, TFMKG and O-KG for their metabolic and functional effects. All three compounds similarly increased the intracellular levels of α−ketoglutarate, yet each of them had multiple effects on other metabolites that were not shared among the three agents, as determined by mass spectrometric metabolomics. While all three compounds reduced autophagy induced by culture in nutrient-free conditions, TFMKG and O-KG (but not DMKG) caused an increase in baseline autophagy in cells cultured in complete medium. O-KG (but neither DMKG nor TFMK) inhibited oxidative phosphorylation and exhibited cellular toxicity. Altogether, these results support the idea that intracellular α-ketoglutarate inhibits starvation-induced autophagy and that it has no direct respiration-inhibitory effect.

## Introduction

α-Ketoglutarate, also known as 2-oxoglutaric acid (IUPAC name: 2-oxopentanedioic acid) is an intermediate of the Krebs cycle, as well as the keto acid produced by deamination of glutamate. In the Krebs cycle, α-ketoglutarate is generated by oxidative decarboxylation of isocitrate (catalyzed by isocitrate dehydrogenase) and then used to generate succinyl coenzyme A (CoA) (catalyzed by α-ketoglutarate dehydrogenase) [1]. During glutaminolysis, glutamine is converted into glutamate and then α-ketoglutarate, which can be introduced into the Krebs cycle as an anaplerotic substrate [2] and is often used in cancer cells (which heavily rely on glutaminolysis) for reductive carboxylation to generate succinate [3].

Macroautophagy (here referred to as ‘autophagy’) is a phylogenetically conserved degradation pathway in which portion of the cytoplasm are wrapped into double-membraned organelles, the autophagosomes, which then fuse with lysosomes for the enzymatic hydrolysis of macromolecules contained in the autophagic cargo into micromolecules that can be used for anabolic reactions or bioenergetic purposes [4,5] Macroautophagy has prominent cytoprotective functions, meaning that it increases the resistance of cells to a variety of external stress signals [6]. In vivo, chronic or cyclic induction of autophagy can cause the extension of lifespan in model organisms including yeast, nematodes, flies and mice [7–9]. Thus, genetic manipulations designed to increase autophagy can increase the health span and longevity of mice [7,10]. Moreover, a few universally effective anti-aging interventions such as caloric restriction [8,11–13], inhibition of mechanistic target of rapamycin complex 1 (MTORC1) [14–16] or supplementation of spermidine [17–20] rely on autophagy, meaning that knockout of essential autophagy genes abolishes their benefits.

Recently, several papers have been published that claim contradictory effects of α-ketoglutarate on autophagy. Several works indicate that addition of the α-ketoglutarate precursor dimethyl α-ketoglutarate (DMKG) to human cell cultures or its intraperitoneal injection into mice effectively inhibits autophagy through an anaplerosis-dependent increase in acetyl-CoA (AcCoA) levels, thus, increasing the acetylation of cytoplasmic proteins [21–25]. In contrast, another paper claims that addition of another α-ketoglutarate precursor, octyl α-ketoglutarate (O-KG) induces autophagy both in human cells and in *Caenorhabditis elegans*, because it inhibits the mitochondrial ATP synthase as well as MTORC1 [26,27].

We hence decided to systematically compare the effects of distinct α-ketoglutarate precursors on cellular metabolism and functions in vitro. Here, we report that three distinct α-ketoglutarate precursors have rather distinct effects and that respiratory chain inhibition and autophagy induction are not universally found in conditions in which intracellular α-ketoglutarate are successfully elevated. Rather, at least in starvation conditions, autophagy inhibition appears to be the universal result of intracellular α-ketoglutarate increases.

## RESULTS and DISCUSSION

### Metabolic effects of α-ketoglutarate precursors

**Figure 1:**
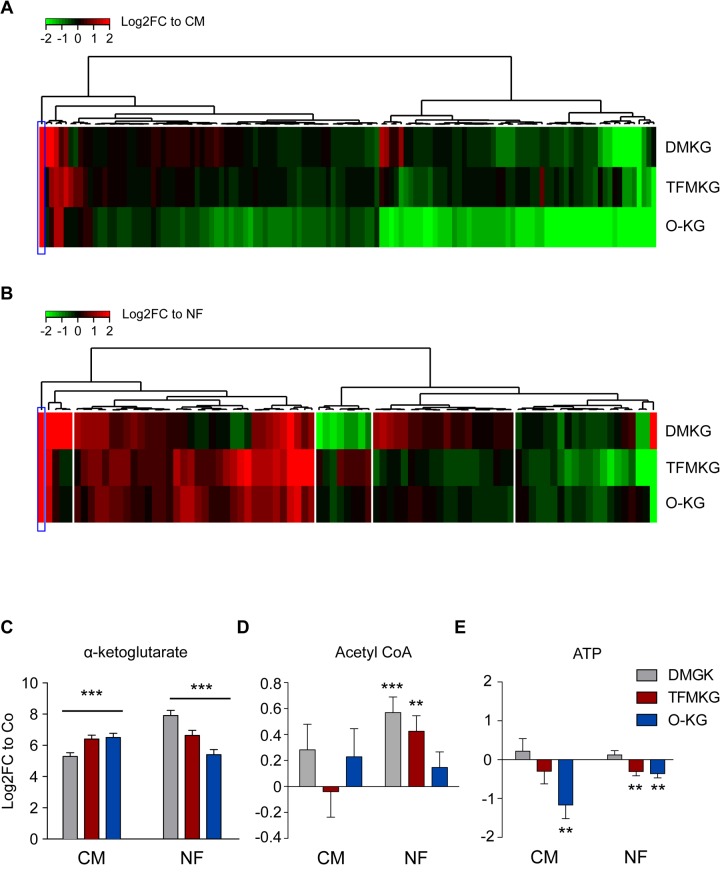
**Metabolic effects of α-ketoglutarate precursors.** (**A, B**) Unsupervised hierarchical clustering of intracellular metabolites in U2OS cells treated with the α-ketoglutarate precursors dimethyl α-ketoglutarate (DMKG), trifluoromethylbenzyl α-ketoglutarate (TFMKG) and octyl α-ketoglutarate (O-KG) in complete (CM) (**A**) or nutrient free (NF) medium (**B**) for 4 h at the concentrations indicated in the Experimental Procedure section. Heat maps depict log_2_ fold changes to the control of metabolite signals found altered (False Discovery Rate [FDR]< 0.1) upon incubation with α-ketoglutarate precursors. (**C-E**) Impact of α-ketoglutarate precursors on intracellular levels of α-ketoglutarate (**C**) and the energy related metabolites AcetylCoA (**D**) and ATP (**E**). Data represent averaged log_2_ fold change ± S.E.M. to the controls (CM or NF). *** p < 0.001; ** p< 0.01 (unpaired *t* test).

Human osteosarcoma cells were cultured in the presence of three distinct precursors of α-ketoglutarate, each of which is a plasma membrane-permeable ester that can be de-esterified by cytosolic esterases to generate α-ketoglutarate, a cell impermeable compound that remains ‘trapped’ within the cell [28]. These three compounds are dimethyl α-ketoglutarate (DMKG), trifluoromethylbenzyl α-ketoglutarate (TFMKG) and octyl α-ketoglutarate (O-KG), which - on theoretical bases - should be hydrolyzed to α-ketoglutarate plus methanol, trifluoromethylphenol and octanol, respectively. Methanol is notoriously membrane-permeable and hence should be readily diffuse from the cell to the medium. To characterize their metabolic effects, we subjected the cells to metabolomic analysis after a 4-hour incubation period in the absence or presence of DMKG, TFMKG or O-KG (all used at two different concentrations described to have biological activity on cultured cells) [21,26,28] by mass spectrometry. This was done both for cells cultured in completed medium (CM), as well as for cells cultured in Hanks’s balanced salt solution (HBSS), which is basically nutrient-free (NF). The latter condition is known to induce autophagy [29]. Unsupervised hierarchical clustering confirmed that the three compounds led to an increase in the intracellular levels of α-ketoglutarate, both in cells cultured in CM (Figure 1A) and in cells cultured in HBSS (Figure 1B). The intracellular raise of α-ketoglutarate was comparable for the three compounds (Figure 1C). Importantly, no other metabolites (apart from α-ketoglutarate and leucylproline) significantly increased in a convergent fashion under the influence of the three α-ketoglutarate precursors when administered in complete medium (Figure 1A,C), as clearly visible from a Venn diagram that visualizes the increase or decrease of different metabolites in CM. (Supplementary Figure 1A, Supplementary Table 1). Of note, the three compounds induced the convergent depletion of four metabolites (NAD^+^, lactoyl-GSH, C6-polyol and β−Alanine) (Supplementary Figure 1A, Supplementary Table 1). When administered in HBSS medium (NF), α-ketoglutarate precursors caused the convergent upregulation of 16 metabolites, while 6 metabolites were found commonly depleted (Supplementary Figure 1B, Supplementary Table 1). Each of the agents induced multiple ‘private’ effects (in the sense that these effects were not shared by any other component) and some of them shared common effects that, however, were not found for the third α-ketoglutarate precursor. Accordingly AcCoA, a metabolite known to stimulate protein acetylation and to inhibit autophagy [21,30] was enhanced by DMKG and TFMKG but not by O-KG in NF conditions (Figure 1D). Adenosine triphosphate levels were reduced by both TFMKG and O-KG but not DKMG (Figure 1E). Altogether, these results demonstrate that the α-ketoglutarate precursors share the ability to increase α-ketoglutarate within cells (the ‘specific’ effect), yet are quite different in their non-specific effects. As a result, we evaluated the functional consequences of DMKG, TFMKG and O-KG in cellular assays on autophagy, respiration and cell survival.

### Inhibition of starvation induced autophagy by α-ketoglutarate precursors

**Figure 2:**
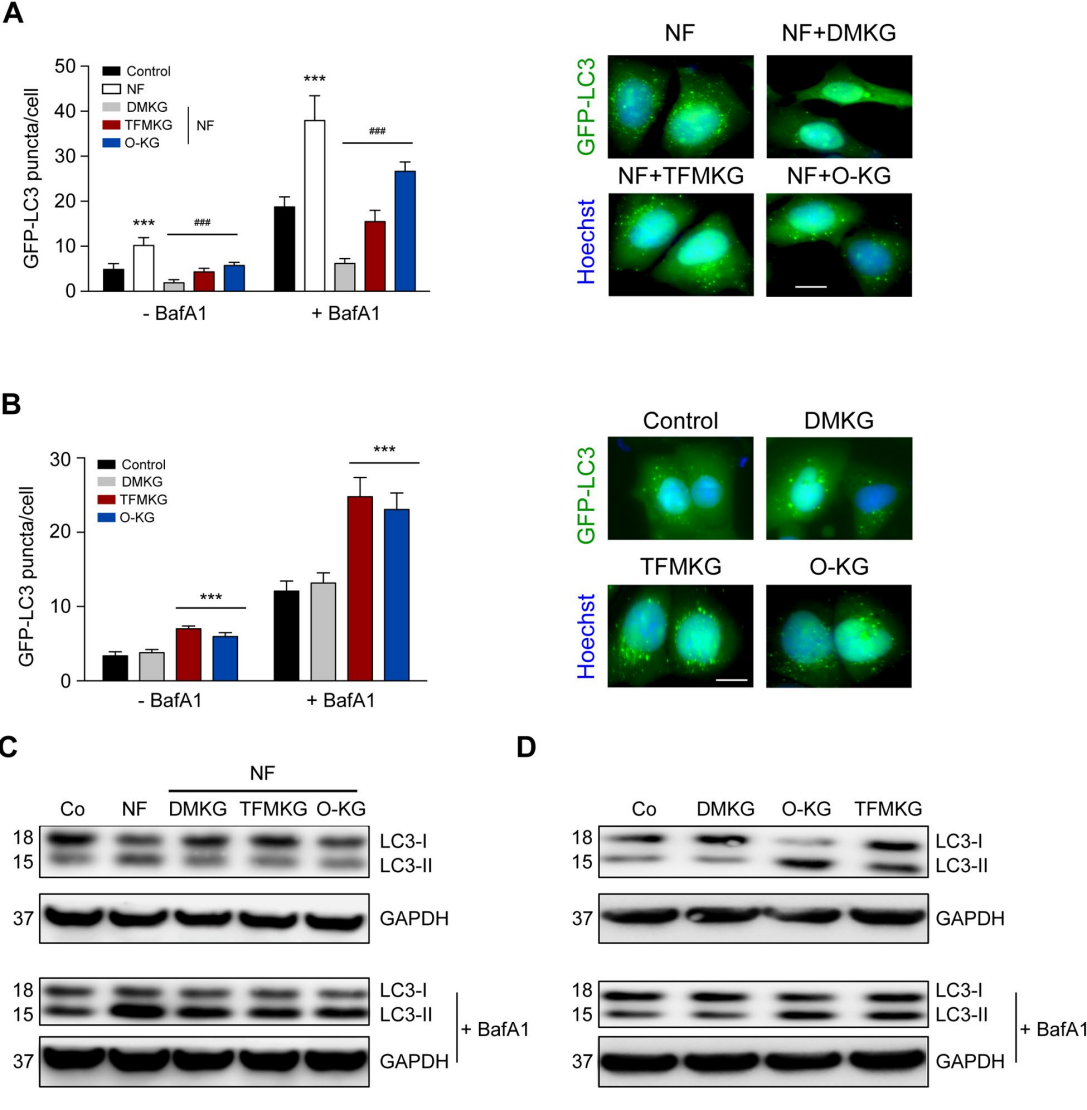
**Modulation of autophagy by α-ketoglutarate precursors.** (**A**) Inhibition of starvation-induced autophagy by DMKG, TFMKG and O-KG. U2OS cells stably expressing the autophagic markers GFP-LC3 were incubated in HBSS medium (NF) and left untreated or incubated with α-ketoglutarate precursors for 4h. Co-treatment with bafilomycin A1 (BafA1) was used to assess autophagic flux. Representative pictures (in presence of BafA1) (right panel) and quantification (left panel) are shown. Data represent mean ± S.D. (one representative experiment, n=3). *** p < 0.001 (compared to Control); ### p < 0.001 (compared to NF) (unpaired *t* test). Scale bar 10 μm. (**B**) Induction of autophagy by TFMKG and O-KG, but not DMKG, in complete medium. *** p < 0.001 (compared to Control); (unpaired *t* test). Scale bar 10 μm. (**C, D**) Immunoblotting showing the conversion of LC3I to LC3II in U2OS cells treated with α-ketoglutarate precursors in NF (**C**) or complete medium (**D**) in presence or absence of BafA1 to monitor autophagic flux (one representative experiment, n=3).

α-Ketoglutarate generated from DMKG inhibits starvation-induced autophagy [12,21,22]. As to be expected, all α-ketoglutarate precursors reduced the number of autophagic puncta per cell in cultures maintained in NF conditions. Such puncta were measured in U2OS cells expressing green fluorescent protein (GFP) fused with microtubule-associated protein 1A/1B-light chain 3B (MAP1LC3B, best known as LC3). In NF conditions, GFP-LC3 puncta increased in the cytoplasm, and this sign of autophagy was suppressed by DMKG, TFMKG and O-KG. In fed conditions (CM), both TFMKG and O-KG, but not DMKG, induced an increase in GFP-LC3 puncta in the presence of bafilomycin A1 (BafA1) as a proof that the two compounds induce autophagic flux (Figure 2A, B).

Similar results were obtained when the membrane redistribution of LC3 was measured by assessing its lipidation that causes an increase in electrophoretic mobility (LC3-II) measurable by immunoblotting. Again, starvation caused the formation of LC3-II, and this effect was largely reduced by prior addition of any of the three cell-permeable α-ketoglutarate precursors. In fed conditions, i.e. when cells were cultured in CM, both TFMKG and O-KG induced immunoblot-detectable autophagy, while DMKG did not enhance LC3-II generation (Figure 2C, D). The NF-induced formation of GFP-LC3 puncta measured in human neuroblastoma H4 cells was also suppressed by DMKG, TFMKG and O-KG (Supplementary Figure 2).

### Compound-specific respiratory effects and toxicity

**Figure 3:**
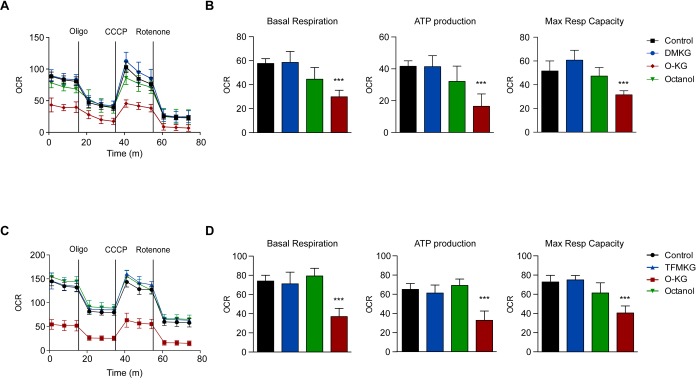
**Influence of α-ketoglutarate precursors on mitochondrial metabolism** (**A-D**) O-KG, but not DMKG and TFMKG, inhibits mitochondrial respiration. U2OS cells were incubated for 6 h in presence or absence of DMKG (**A, B**), TFMKG (**C, D**), O-KG and octanol (**A-D**); after pre-incubation with distinct α-ketoglutarate precursors, oxygen consumption rate (OCR) was monitored in a Seahorse XF analyzer upon injection of the complex V inhibitor oligomycin (Oligo), the uncoupler carbonyl cyanide 3-chlorophenylhydrazone (CCCP) and the complex I inhibitor rotenone at the concentrations indicated in the Experimental Procedure section. Mitochondrial function was evaluated as basal respiration (**B, D,** left panel), ATP production (**B, D,** middle panel) and maximal respiratory capacity (**B, D,** right panel). Data are depicted as mean ± S.D. (one representative experiment, n=3). *** p < 0.001 (compared to Control) (unpaired *t* test)

**Figure 4:**
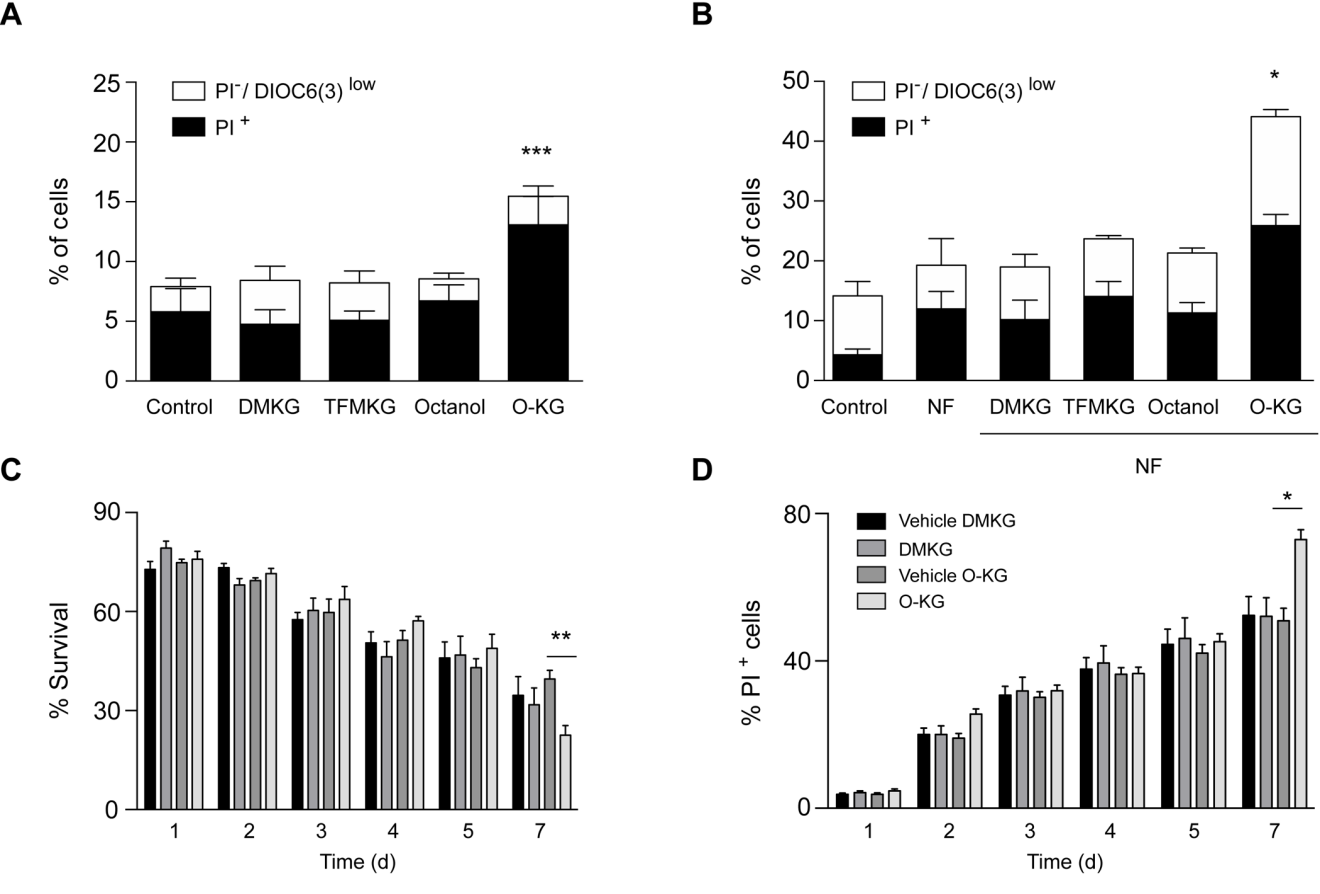
**Impact of α-ketoglutarate precursors on cell viability.** (**A-B**) Cytofluorimetric assessment of cell death elicited upon administration of distinct α-ketoglutarate precursors to U2OS cells in complete (**A**) or nutrient free medium (**NF**) (**B**) for 4 h. PI^+^ = dead cells; PI^-^/DiOC_6_(3) low cells = dying cells. Data (depicted as percentage of cells) represent mean ± S.D. (one representative experiment, n=3). *** p < 0.001 (compared to Control); * p < 0.05 (compared to NF) (unpaired *t* test). (**C**). Survival rates of treated (200 µM) and control cells were analyzed at indicated timepoints via clonogenicity assay. (**D**) Plasma membrane integrity via PI staining of treated (200 µM) versus control yeast cells was monitored at indicated timepoints during chronological aging. Data represent mean ± S.E.M of at least 3 independent experiments. ** p < 0.01; * π < 0.05 (Compared to O-KG vehicle); (two-way Anova).

We next determined the capacity of DMKG, TFMKG and O-KG to affect oxidative phosphorylation by means of a Seahorse analyzer. While DMKG and TFMKG failed to affect respiration, O-KG markedly reduced basal and maximal respiration, as well ATP production in a concentration-dependent manner (Figure 3). Of note, octanol had no effect on respiration. This differential effect correlated with the level of cytotoxicity determined by staining cells with a combination of the mitochondrial transmembrane potential probe 3,3′-dihexyloxacarbocyanine iodide (DiOC_6_(3)) and the vital dye propidium iodide (PI) to detect the percentage of dying (DiOC_6_(3)^low^ PI^-^) and dead (DiOC_6_(3)^low^ PI^+^) cells [31,32]. DMKG, TFMKG or octanol all failed to induce cell death and hence were compatible with cellular survival, whereas O-KG showed enhanced toxicity (Figure 4A, B). Consistent with this data, administration of O-KG (but not DMKG) to *S. cerevisiae* during chronological aging progressively impaired survival (Figure 4C) and reduced viability (Figure 4D) of yeast cells.

### Concluding remarks

It has been claimed by several independent groups that α-ketoglutarate inhibits autophagy [21–25,33]. However, the contrary has been reported as well, namely, that α-ketoglutarate would induce autophagy [26]. This contradiction can be most probably explained by major methodological differences, in particular the use of distinct α-ketoglutarate precursors. DMKG and TFMKG both cause autophagy inhibition in NF conditions [12,21–24,33]. In sharp contrast, O-KG (which, at difference with DMKG and TFMKG, does inhibit respiration) may induce autophagy in baseline conditions, without starvation [26]. Oral administration of α-ketoglutarate (1 or 2% in drinking water) to mice, which however should not be bioavailable as intracellular α-ketoglutarate, also reportedly inhibits autophagy [34]. DMKG and TFMKG are also both known to inhibit the hypoxia-induced activation of hypoxia-inducible factor-1α (HIF-1α), but this effect was not confirmed for O-KG [33,35].

What might be the mechanism accounting for these discrepancies? In our study, we found that DMKG, TFMKG and O-KG similarly inhibited starvation-induced autophagy, while TFMKG and O-KG (but not DMKG) caused a small increase in autophagic flux (though not comparable to the effect of starvation) in cells cultured in CM. Importantly, it remains to be established whether ‘private’ metabolites generated upon the administration of distinct α−ketoglutarate precursors can engender differential effects on autophagy induction. Of note, we found that TFMKG and O-KG caused a reduction in ATP levels, in fed cells, while this was not found for DMKG. This may explain differential autophagy induction. As reported [26], O-KG inhibited oxidative phosphorylation, but both DMKG and TFMKG failed to mediate such an effect. Since the three compounds cause a similar increase in cellular α−ketoglutarate levels, this suggests that α-ketoglutarate may not be responsible for respiratory inhibition. Theoretically, it might be possible that the three compounds are metabolized in a differential fashion with a differential access to the mitochondrial matrix. However, this possibility appears remote because the esterase(s) liberating α-ketoglutarate from its synthetic precursors should be the same, with a similar subcellular redistribution. While TFMKG reduces ATP levels in fed cells, it does not inhibit respiration. Hence, the mechanism through which TMFKG might cause this effect remains to be explored. O-KG was the sole agent that was markedly cytotoxic on yeast and human cells, contrasting with the absent toxicity of DMKG and TFMKG. It is tempting to speculate that this effect might be explained by the mitochondrial toxicity of the compound, knowing that mitochondrial are central in cell death signaling [36–38]. As a possibility, O-KG might gain access to the mitochondrial matrix before it is hydrolyzed to octanol and α-ketoglutarate and then mediate a respiratory chain-inhibitory effect that is not shared by α-ketoglutarate.

In sum, our data suggest that several effects that have been attributed to α-ketoglutarate are caused by the precursor of α-ketoglutarate that is administered rather than by α-ketoglutarate itself. Of note, octanol did not recapitulate any of the effects of O-KG, suggesting that it is O-KG itself (or another yet-to-be-discovered degradation product of O-KG) rather than α-ketoglutarate itself that accounts for its mitochondriotoxic and cytotoxic effects. At this stage, it appears clear that the universal claim of α-ketoglutarate would be an autophagy inducer and an inhibitor of mitochondrial respiration must be revised.

## MATERIALS AND METHODS

### Chemicals and culture conditions

Unless otherwise indicated, media and supplements for cell culture were purchased from Gibco-Invitrogen Life Technologies Inc. (Carlsbad, CA, USA) while plasticware was purchased from Corning B.V. Life Sciences (Amsterdam, The Netherlands). Human osteosarcoma U2OS, human neuroglioma H4 and their green fluorescent protein (GFP)-LC3-expressing derivatives were cultured in DMEM medium supplemented with 10% (v/v) fetal bovine serum, 100 mg/L sodium pyruvate, 10 mM HEPES buffer, 100 IU mL-1 penicillin G sodium salt, and 100 mg/mL streptomycin sulfate. All cells were maintained in standard culture conditions (at 37° C, under 5% CO_2_). Cells were seeded in 6- or 96- wells plates before 4 h treatment with 5 mM dimethyl 2-ketoglutarate (349631, Sigma Aldrich), 2 mM trifluoromethylbenzyl α-ketoglutarate (gift from Pr. Gottlieb), 1 mM octyl α-ketoglutarate (11970, Cayman Chemicals) or 1 mM 1-octanol (297887, Sigma Aldrich) in complete DMEM medium (CM) or in HBSS medium (NF). To assess autophagic flux, 100 nM bafilomycin A1 (B1080, LC Laboratories) was added 1 h before cells harvesting.

### Flow cytometry

The following fluorochromes were employed to determine apoptosis-associated modifications: 3,39-dihexyloxacarbocyanine iodide (DiOC_6_(3), 40 nM) for quantification of the mitochondrial transmembrane potential and propidium iodide (PI; 1 μg/ml) (both from Molecular Probes) for determination of cell viability. U2OS cells, treated as mentioned before, then were trypsinized and labeled with the indicated fluorochromes at 37°C, followed by cytofluorometric analysis by Attune flow cytometer (Thermo Fisher Scientific).

### Immunoblotting

For immunoblotting, protein extracts obtained by cellular lysis in radioimmunoprecipitation assay (RIPA) buffer were run on 4-12% bis-tris acrylamide gels (Thermo Fisher) and electrotransferred to 0.2 μM polyvinylidene fluoride (PVDF) membranes (Bio-Rad) Non-specific binding sites were saturated by incubating membranes for 1 h in 0.05% Tween 20 (v:v in Tris-buffered saline, TBS) supplemented with 5% non-fat powdered milk (w:v in TBS), followed by an overnight incubation with primary antibodies specific for LC3 (#2775, Cell Signaling Technology). Equal loading was monitored by probing membranes with a glyceraldeyde-3-phosphate dehydrogenase (GAPDH)-specific antibody (#2118, Cell Signaling Technology). Membranes were developed with suitable horseradish peroxidase conjugates (Southern Biotechnologies), followed by chemiluminescence-based detection with the SuperSignal West Pico® reagent (Thermo Scientific) and the ImageQuant LAS 4000 software-assisted imager (GE Healthcare).

### Metabolomics

### *Cell preparation*

Cells were cultured in 6-well plates being at an approximate 80% of confluence the day of the experiment. After the corresponding treatment, wells plates were placed upon ice under chemical hood and processed. Wells were softly and quickly (<2s) washed with cold milliQ water (+4°C). Wells were then lysed with 500µl of cold methanol/water (9/1, v/v, -20°C, with internal standards), scrapped and pooled (2 wells per conditions) in microcentrifuge tubes. Cold chloroform (100µl, -20°C) was added to the lysate. Solution was vortexed for 30s and centrifuged at 15000 rpm for 10 min at +4 °C. Supernatant was collected and evaporated in microcentrifuge tubes at 40°C in a pneumatically-assisted concentrator (Techne DB3). 300µl of methanol was added on dried extract and split in two parts of 150µl: the first one used for the GC-MS experiment, the second one used for the LC-MS analysis.

### *Untargeted analysis of intracellular metabolites by UHPLC coupled to a quadrupole- time of flight (QTOF) mass spectrometer*

Profiling of intracellular metabolites was performed on a RRLC 1260 system (Agilent Technologies, Waldbronn, Germany) coupled to a QTOF 6520 (Agilent) equipped with an electrospray source operating in both positive and negative mode and full scan mode, from 50 to 1000Da. The gas temperature was set at 350°C with a gas flow of 12l/min. The capillary voltage was set at 3.5kV, and the fragmentor at 120V. Two reference masses were used to maintain the mass accuracy during analysis: m/z 121.050873 and m/z 922.009798 in positive mode and m/z 112.985587 and m/z 980.016375 in negative mode. 10μL of sample were injected on a SB-Aq column (100mm × 2.1mm, particle size 1.8μm) from Agilent Technologies, protected by a guard column XDB-C18 (5mm × 2.1mm, particle size 1.8μm) and heated at 40°C. The gradient mobile phase consisted of water with 0.2% of acetic acid (A) and acetonitrile (B). The flow rate was set to 0.3 ml/min. Initial condition is 98% phase A and 2% phase B. Molecules are then eluted using a gradient from 2% to 95% phase B in 7 min. The column was washed using 95% mobile phase B for 3 minutes and equilibrated using 2% mobile phase B for 3 min. The autosampler was kept at 4°C.

### *Targeted analysis of intracellular metabolites by UHPLC coupled to a Triple Quadrupole (QQQ) mass spectrometer*

Targeted analysis was performed on a RRLC 1260 system (Agilent) coupled to a Triple Quadrupole 6410 (Agilent) equipped with an electrospray source operating in positive mode. The gas temperature was set at 350°C with a gas flow of 12l/min. The capillary voltage was set at 3.5kV. 10μL of sample were injected on a Column Zorbax Eclipse plus C18 (100mm x 2.1mm, particle size 1.8μm) from Agilent technologies, protected by a guard column XDB-C18 (5mm × 2.1mm, particle size 1.8μm) and heated at 40°C. The gradient mobile phase consisted of water with 2mM of DBAA (A) and acetonitrile (B). The flow rate was set to 0.2 ml/min, and gradient as follow: initial condition is 90% phase A and 10% phase B, maintained during 4 min. Molecules are then eluted using a gradient from 10% to 95% phase B over 3 min. The column was washed using 95% mobile phase B for 3 minutes and equilibrated using 10% mobile phase B for 3 min. The autosampler was kept at 4°C. The collision gas was nitrogen. The scan mode used was the MRM for biological samples. Peak detection and integration of the analytes were performed using the Agilent Mass Hunter quantitative software (B.07.01).

### *Widely-targeted analysis of intracellular metabolites gas chromatography (GC) coupled to a triple quadrupole (QQQ) mass spectrometer*

The GC-MS/MS method was performed on a 7890A gas chromatography (Agilent) coupled to a triple quadrupole 7000A (Agilent) equipped with a High sensitivity electronic impact source (EI) operating in positive mode. The front inlet temperature was 250°C, the injection was performed in splitless mode. The transfer line and the ion-source temperature were 250°C and 230°C, respectively. The septum purge flow was fixed at 3 mL/min, the purge flow to split vent operated at 80 mL/min during 1 min and gas saver mode was set to 15 mL/min after 5 min. The helium gas flowed through the column (J&WScientificHP-5MS, 30m x 0.25 mm, i.d. 0.25 um, d.f., Agilent Technologies Inc.) at 1 mL/min. Column temperature was held at 60°C for 1 min, then raised to 210°C (10°C/min), followed by a step to 230°C (5°C/min) and reached 325°C (15°C/min), and be hold at this temperature for 5 min. The collision gas was nitrogen. The scan mode used was the MRM for biological samples. Peak detection and integration of the analytes were performed using the Agilent Mass Hunter quantitative software (B.07.01).

### Automated microscopy

Cell stably expressing GFP-LC3 were seeded in 96-well or 384-well imaging plates (BD Falcon) in complete (CM) or nutrient free (NF) medium. Cells were treated with α-ketoglutarate precursors for 4 hours. Bafilomycin A1 was added 1 hour before fixation. Subsequently, cells were fixed with 4% PFA and counterstained with 10 μM Hoechst 33342. Images were acquired using a BD pathway 855 automated microscope (BD Imaging Systems) equipped with a 40X objective (Olympus) coupled to a robotized Twister II plate handler (Caliper Life Sciences). Images were analyzed for the detection of GFP-LC3 puncta in the cytoplasm by means of the BD Attovision software (BD Imaging Systems). Cellular regions of interest, cytoplasm and nucleus, were defined and segmented according to standard procedures. RB 2x2 and Marr-Hildreth algorithms were employed to allow the detection of GFP LC3 puncta. Statistical analyses were conducted using Prism software.

### Analysis of mitochondrial metabolism

Cellular respiration was measured using the XF-96 analyzer (Seahorse Bioscience). Mitochondrial bioenergetic assays were performed according to manufacturer’s instructions. The XF assay medium (Seahorse Bioscience) was supplemented with 4 mM L-glutamine, 1 mM pyruvate, and 1 g/l D-glucose and pH was adjusted with 1 M NaOH to 7.4 at 37°C. Ten x 10^3^ cells were seeded per well and allowed to adapt for 24 h to obtain a monolayer of cells before measurement. After 4 hours treatment with α-ketoglutarate precursors, basal respiration was measured; mitochondrial respiration test was performed by sequential additions of 1 μM oligomycin, 0.5 μM carbonyl cyanide-4-(trifluoromethoxy)phenylhydrazone (CCCP) and 1 μM rotenone. Maximal respiration induced by CCCP uncoupler was corrected by subtracting the non-mitochondrial respiration values. Subsequently the cells were fixed with 3.7% of PFA supplemented with 1 μM Hoechst 33342 for 20 min. PFA was substituted with PBS and whole-well imaging was performed by means of BD pathway 855 automated microscope.

### Yeast chronological aging, clonogenicity and cell death assay

The yeast wild type strain BY4742 (MATα *his3Δ1 leu2Δ0 lys2Δ0 ura3Δ0*) obtained from Euroscarf was used for all experiments. Yeast cells were grown in SC medium containing 0.17% yeast nitrogen base (BD Diagnostics, Schwechat, Austria), 0.5% (NH_4_)_2_SO_4_, 30 mg/l of all amino acids (except 80 mg/l histidine and 200 mg/l leucine), 30 mg/l adenine and 320 mg/l uracil, with 2% glucose (SCD). Chronological aging experiments were performed in 96-deepwell plates, using 500 µl of freshly inoculated medium (OD_600_ of 0.1; ~1x10^6^ cells/ml) per well. Cells were grown to an OD_600_ of ~0.2 and supplemented with either 200 µM of the indicated compound (dimethyl-α-ketoglutarate, obtained from Sigma Aldrich, Germany; octyl-α-ketoglutarate obtained from Biomol, Germany) or the respective solvent (ddH_2_O or methanol, reaching a final concentration of 0.2%) as a control. At indicated timepoints membrane dysintegrity as a marker of cell death was assayed using propidium iodide (PI) staining as described [39] and PI positive cells were quantified by flow cytometry (BD LSRII Fortessa, BD Biosciences). Clonogenicity assays were performed as described [39]. In short, cell counts of treated and control cultures were measured with a CASY cell counter (Schärfe system GmbH) and 500 cells were plated on YPD agar plates. For calculating the survival rate, colony-forming units (CFU) were analyzed after two days of incubation at 28°C using an automated cell counter (LemnaTech).

## Supplementary Material

Supplementary Figures

Supplementary Table
