# Supplementary material for: α-Ketoglutarate inhibits autophagy
Source: Aging (Albany NY). 2019 Jun 7;11(11):3418–31. doi: 10.18632/aging.102001 (PMC6594794; doi:10.18632/aging.102001)
Supplement: Supplementary Figures [file aging-11-102001-s002.pdf]

SUPPLEMENTARY FIGURES

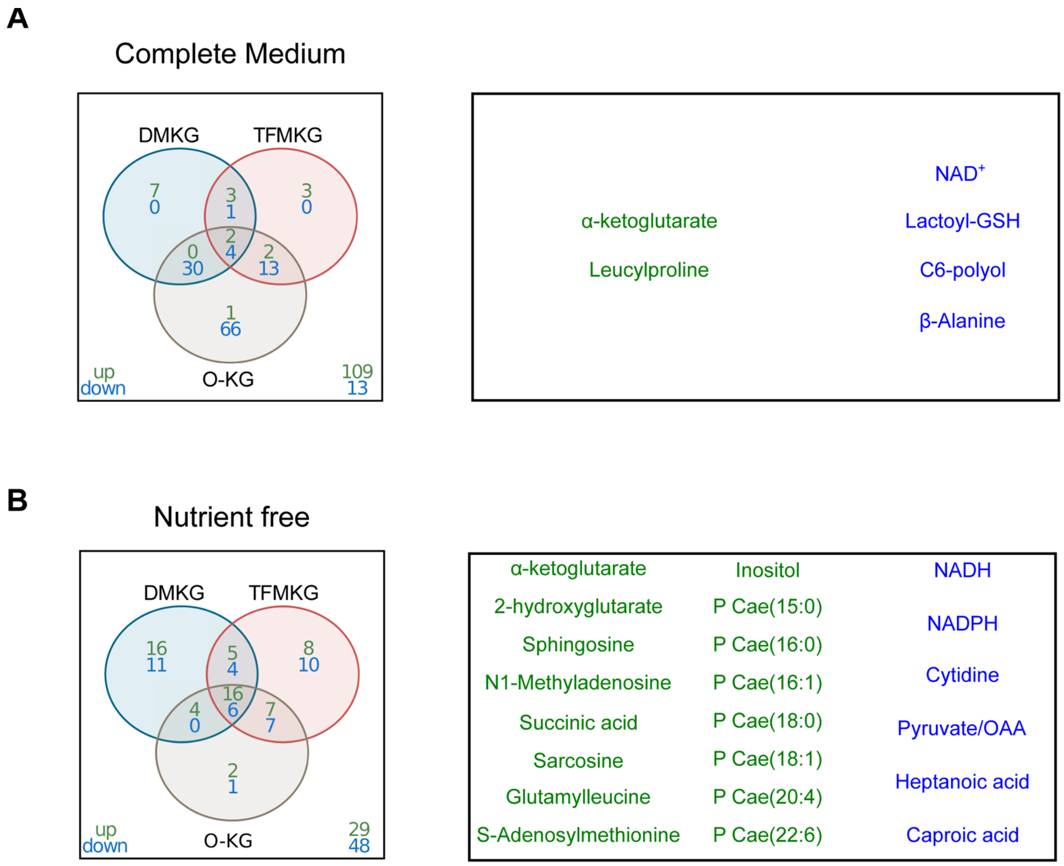

Supplementary Figure 1 (related to Figure 1). Shared *vs.* private effects of α-ketoglutarate precursors on intracellular metabolome. Venn diagrams depicting the number of metabolites (FDR < 0.1) up- or down- regulated upon administration of α-ketoglutarate precursors in complete medium (**A**, left panel) or nutrient free medium (**B**, left panel). Metabolites that are convergently modulated (p < 0.05) by the three compounds are indicated in **A** and **B** (right panels).

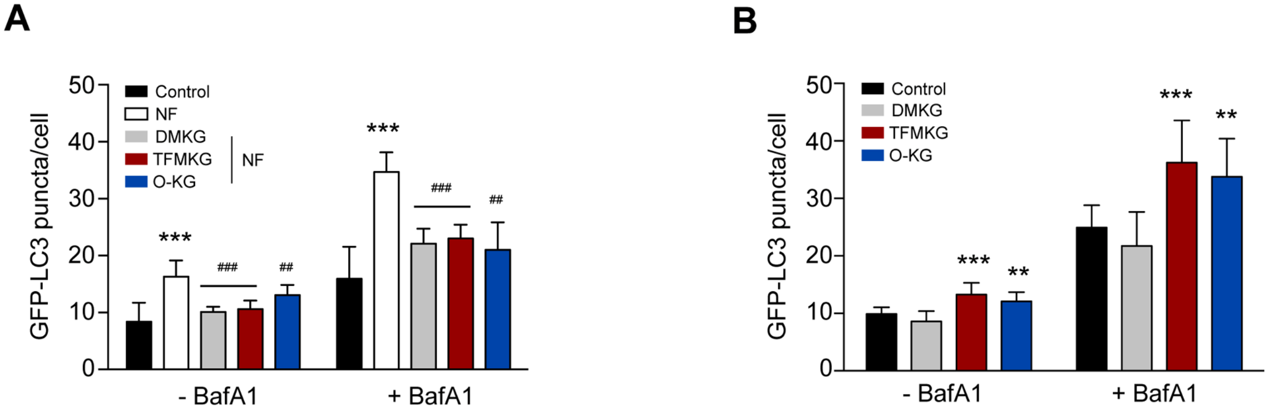

Supplementary Figure 2 (related to Figure 2). Modulation of autophagy by α-ketoglutarate precursors in H4 cells. (A) Inhibition of starvation-induced autophagy by DMKG, TFMKG and O-KG. H4 cells stably expressing the autophagic markers GFP-LC3 were incubated in HBSS medium (NF) and left untreated or incubated with α-ketoglutarate precursors. Co-treatment with Bafilomycin A1 (BafA1) was used to assess autophagic flux. Data represent mean ± S.D. (one representative experiment, n=3). *** p < 0.001 compared to Control; ## p < 0.01, ### p < 0.001 compared to NF (unpaired *t* test). **(B)** Induction of autophagy by TFMKG and O-KG, but not DMKG, in complete medium. ** p < 0.01, *** p < 0.001 compared to Control (unpaired *t* test).
